# Association of Lifestyle Factors and Antihypertensive Medication Use With Risk of All-Cause and Cause-Specific Mortality Among Adults With Hypertension in China

**DOI:** 10.1001/jamanetworkopen.2021.46118

**Published:** 2022-02-01

**Authors:** Qi Lu, Yanbo Zhang, Tingting Geng, Kun Yang, Kunquan Guo, Xinwen Min, Meian He, Huan Guo, Xiaomin Zhang, Handong Yang, Tangchun Wu, An Pan, Gang Liu

**Affiliations:** 1 Department of Nutrition and Food Hygiene, Hubei Key Laboratory of Food Nutrition and Safety, Ministry of Education Key Laboratory of Environment and Health, and State Key Laboratory of Environmental Health (Incubating), School of Public Health, Tongji Medical College, Huazhong University of Science and Technology, Wuhan, China; 2 Department of Epidemiology and Biostatistics, Ministry of Education Key Laboratory of Environment and Health, School of Public Health, Tongji Medical College, Huazhong University of Science and Technology, Wuhan, China; 3 Department of Endocrinology, Affiliated Dongfeng Hospital, Hubei University of Medicine, Shiyan, China; 4 Department of Occupational and Environmental Health, Ministry of Education Key Laboratory of Environment and Health, School of Public Health, Tongji Medical College, Huazhong University of Science and Technology, Wuhan, China; 5 Sinopharm Dongfeng General Hospital, Hubei University of Medicine, Shiyan, China

## Abstract

**Importance:**

The joint association of antihypertensive medication use and healthy lifestyle with mortality among individuals with hypertension is unclear.

**Objective:**

To examine the association of lifestyle factors combined with antihypertensive medication use, as well as changes in lifestyle, with all-cause and cause-specific mortality among individuals with hypertension.

**Design, Setting, and Participants:**

This cohort study used data from the Dongfeng-Tongji cohort, a long-term, prospective cohort including employees at a manufacturer in China, with baseline from 2008 to 2010. Participants with hypertension were followed up for a median (IQR) of 7.3 (5.7-10.3) years, ending in 2018. Data were analyzed from February to April 2021.

**Exposures:**

Lifestyle factors, including body mass index, smoking status, diet, physical activity, and sleep duration, were coded on a 3-point scale (range, 0-2, with higher score indicating a healthier lifestyle). Lifestyle was evaluated according to the total score of all 5 factors, and categorized into 3 groups: unfavorable (scores 0-4), intermediate (scores 5-7), and favorable (scores 8-10). Antihypertensive medication use was defined as use within the last 2 weeks.

**Main Outcomes and Measures:**

All-cause, cardiovascular, and cancer mortality were identified by linking the cohort database with the health care system through December 31, 2018.

**Results:**

A total of 14 392 participants (mean [SD] age, 65.6 [7.4] years; 7277 [50.6%] men and 7115 [49.4%] women) with hypertension were included, and 2015 deaths were documented, including 761 cardiovascular deaths and 525 cancer deaths. Compared with individuals not using antihypertensive medication and with a lifestyle score of 0 to 4, the combination of using antihypertensive medication and having a lifestyle score of 8 to 10 was associated with the lowest risk of all-cause mortality (hazard ratio [HR], 0.32; 95% CI, 0.25-0.42), cardiovascular mortality (HR, 0.33; 95% CI, 0.21-0.53), and cancer mortality (HR, 0.30; 95% CI, 0.19-0.47). In addition, improvement in lifestyle score after hypertension diagnosis was associated with lower risk of all-cause mortality (HR, 0.52; 95% CI, 0.36-0.76) and cardiovascular mortality (HR, 0.53; 95% CI, 0.30-0.94).

**Conclusions and Relevance:**

These findings suggest that adherence to healthy lifestyle and antihypertensive medication treatment were associated with lower risk of mortality among adults with hypertension. These findings further support that, in addition to antihypertensive medication use, adopting a healthy lifestyle is associated with benefits in the prevention of premature death among individuals with hypertension.

## Key Points

**Question:**

Are the combination of antihypertensive medication use and healthy lifestyle, as well as the change in lifestyle, associated with mortality among individuals with hypertension?

**Findings:**

In this cohort study of 14 392 individuals with hypertension, adherence to healthy lifestyle and antihypertensive medication treatment was associated with lower risk of all-cause, cardiovascular, and cancer mortality. Improvement in lifestyle after hypertension diagnosis was also associated with significantly lower risk of mortality.

**Meaning:**

These findings suggest that in addition to antihypertensive medication use, adopting a healthy lifestyle is associated with benefits in the prevention of premature death among individuals with hypertension.

## Teaser

This cohort study assesses the association of lifestyle factors and antihypertensive medication use with risk of mortality among individuals with hypertension in China.

## Introduction

Hypertension is a major public health concern, affecting 1.13 billion adults worldwide.^1^ Despite the considerable advances in antihypertensive medication treatments, the prevalence of hypertension has been increasing over the past 40 years.^2^ Elevated blood pressure (BP) is a leading cause of cardiovascular disease (CVD) and mortality^3^ and accounts for more than 10 million deaths in 2019.^4^ It is paramount to identify effective strategies to prevent or delay the poor prognosis for individuals with hypertension.

Adherence to antihypertensive medication treatment and following a healthy lifestyle are important parts of hypertension self-management.^5^ Nonadherence to medication regimen has been reported in 10% to 80% of patients with hypertension, with higher rates in low-income countries and rural areas,^6,7^ and is an essential risk factor for nonoptimal BP control and cardiovascular events.^8,9^ Maintaining a healthy lifestyle can improve vascular health, prevent or delay the onset of hypertension, and is associated with lower cardiovascular risk.^10,11,12^ Moreover, it is suggested that lifestyle modification may be more important than medication treatment for mild hypertension.^13^ However, the evidence regarding the long-term health outcomes associated with the combination of antihypertensive medication use and healthy lifestyle among individuals with hypertension is limited. Although epidemiologic studies have suggested that individuals with hypertension who adopted a relatively healthy lifestyle but did not use antihypertensive medications had a lower risk of stroke or heart failure, compared with those who used antihypertensive medications but did not adhere to a healthy lifestyle,^14,15^ no study has examined the joint association of antihypertensive medication use and healthy lifestyle with mortality among individuals with hypertension, to our knowledge. In addition, whether improvement in lifestyle may yield health benefits for individuals with hypertension is unclear.

To fill these knowledge gaps, we aimed to prospectively examine the association of antihypertensive medication use and healthy lifestyle, as well as changes in lifestyle, with risk of all-cause and cause-specific mortality among middle-aged and elderly adults with hypertension in China.

## Methods

This cohort study was approved by the Medical Ethics Committee of the School of Public Health, Tongji Medical College, Huazhong University of Science and Technology and Dongfeng General Hospital, Dongfeng Motor Corporation (DMC), Shiyan City, China. All participants provided written informed consent. Our study follows the Strengthening the Reporting of Observational Studies in Epidemiology (STROBE) reporting guideline.

### Study Population

The Dongfeng-Tongji (DFTJ) study is an ongoing dynamic cohort that consecutively recruits retired employees of DMC.^16^ The DFTJ study was launched in 2008, and 2 follow-up surveys have been completed since. Briefly, 27 009 participants were recruited at baseline from September 2008 to June 2010. Among these, 25 978 individuals (96.2%) were followed up in 2013; meanwhile, 14 120 new retirees were additionally enrolled into the cohort. In 2018, we completed the second follow-up survey. The detailed timeline is presented in eFigure 1 in the Supplement. Face-to-face interviews, physical examinations, and blood draws were conducted at all surveys.

This study included 17 308 participants with prevalent hypertension, defined as self-reported physician-diagnosed hypertension or use of antihypertensive medication within the last 2 weeks (including 9719 participants from 2008, 3875 participants from 2008-2013, and 3714 participants from 2013). After excluding 1031 participants with cancer at baseline and those with incomplete information on antihypertensive medication use (60 participants) or any components of lifestyle (1825 participants), a total of 14 392 participants with hypertension were included in the final analysis (eFigure 1 in the Supplement).

### Assessment of Lifestyle Factors

Lifestyle factors assessed included body mass index (BMI; calculated as weight in kilograms divided by height in meters squared), smoking status, diet, physical activity, and sleep duration. BMI was grouped into 3 categories as reference range (18.5-24.9), overweight (25.0-29.9), and obese or underweight (≥30.0 or <18.5).^17^ According to previous literature,^1^ underweight was associated with increased mortality rate among older adults at a higher rate even than overweight. Thus, in our analysis, overweight was categorized as intermediate lifestyle, and underweight and obese were grouped into unfavorable lifestyle. In addition, among 14 392 participants included in the current analysis, only 216 (1.5%) were underweight. In a sensitivity analysis, we also used the Asian BMI cutoff (ie, overweight: ≥24.0; obese: ≥28.0). Smoking status was grouped into noncurrent smokers (never smoking or having quit >10 years), former smokers having quit 10 years or less, and current smokers.

Information on the frequency and mean duration of leisure-time physical activity, including walking, jogging, biking, playing ball games, dancing, tai chi, swimming, exercising at the gym, and others, was collected through questionnaire. Metabolic equivalents (METs) were used to define moderate (3 to <6 METs) and vigorous (≥6 METs) activities, such as 3 METs for walking, 4 METs for biking, 4.5 METs for tai chi, 5 METs for dancing or calisthenics, 6 METs for playing ball games or exercising at the gym, and 7.5 METs for jogging and swimming. The optimal level of physical activity was defined as weekly moderate activities for at least 150 minutes or weekly vigorous activities for at least75 minutes. Intermediate level of physical activity referred to any level of moderate or vigorous activities greater than 0 but not reaching optimal level. Physical inactivity was considered as no moderate or vigorous activities.

Diet was assessed based on 3 food components, including vegetables, fruits, and meat, that were addressed in the 2013 American Heart Association guideline on lifestyle management to reduce cardiovascular risk.^18^ Participants were assigned 1 point for each if they consumed vegetables at least twice a day, fruits at least once a day, or meat less than once a day; otherwise, 0 points. Diet quality was graded according to the total score: favorable (3 points), intermediate (2 points), and unfavorable (0-1 point).

According to the *J*-shaped association between sleep duration and all-cause mortality reported in previous literature,^19^ nighttime sleep duration was divided into 3 groups: optimal (6-8 hours/day), intermediate (5-5.9 or 8.1-10 hours/day), and poor (<5 or >10 hours/day).

### Definition of Overall Favorable Lifestyle

Overall favorable lifestyle was evaluated based on the aforementioned 5 lifestyle factors, which have been highlighted in recent guidelines in the prevention and treatment of hypertension.^7^ We coded 2 points to participants for each low-risk factor: BMI within reference range, noncurrent smoking status, engaging in optimal level of physical activity, favorable diet, and optimal sleep duration. Participants with high-risk factors were coded 0 points, including those who were obese or underweight, current smoking status, physical inactivity, unfavorable diet, and poor sleep duration. The remaining lifestyle factors were assigned 1 point. Overall lifestyle score was the sum of the individual scores of all 5 lifestyle factors, ranging from 0 to 10, with a higher score indicating a healthier lifestyle. Participants were categorized into 3 groups using the cutoff values of lifestyle score that were most practical and with sufficient statistical power in each category: favorable (8-10 points), intermediate (5-7 points), and unfavorable (0-4 points) lifestyle.

### Assessment of Antihypertensive Medication Use and Other Covariates

Use of antihypertensive medication was classified as responding yes to the question *Have you used any antihypertensive medications within the last 2 weeks?* Other covariates, including age, sex, education attainment, drinking status, hypertension duration, self-reported physician-diagnosed diabetes or CVD (including coronary heart disease, myocardial infraction, and stroke), and use of hypoglycemic and lipid-lowering medication, were obtained via questionnaires. Mental stress (available only in the first survey) was defined according to 7 yes or no questions about stress and mental status in the past 1 month, ie, losing interest, feeling tired or low on energy, weight change, sleep disorders, difficult concentrating, thought about death, or feeling worthless. Participants were categorized into 3 groups: with 3 or more symptoms, 1 to 2 symptoms, or no symptoms.

In addition, BP was measured by trained investigators according to standard procedures. Metabolic biomarkers, including fasting glucose, glycated hemoglobin A_1c_, total cholesterol, triglyceride, high-density lipoprotein (HDL) cholesterol, low-density lipoprotein cholesterol, and creatinine levels, were measured among the participants who provided blood samples at baseline and follow-up surveys. The details are documented elsewhere.^16^ Estimated glomerular filtration rate was computed based on the Chronic Kidney Disease Epidemiology Collaboration equation.^20^

### Ascertainment of Mortality

Vital status was confirmed by linkage with the health care system of DMC that archived death certificates of all retirees through December 31, 2018. The *International Statistical Classification of Diseases and Related Health Problems, Tenth Revision (ICD-10) *was used to define cardiovascular deaths (*ICD-10* codes I00-I99) and cancer deaths (*ICD-10* codes C00-C97).

### Statistical Analysis

#### Basic Information

Participants were categorized into 6 groups according to different combinations of antihypertensive medication use (yes or no) and overall lifestyle (unfavorable, intermediate, and favorable). Baseline characteristics were described across groups. Person-years were calculated from the date responding to questionnaires to the date when death occurred or the end of follow-up (December 31, 2018), whichever came first. Missing values of covariates (<4.4%) were imputed with median for continuous variables and missing indicators for categorical variables.

#### Association of Lifestyle and Antihypertensive Medication Use With Mortality

Cox proportional hazards regression models were used to examine the joint associations of antihypertensive medication use and lifestyle with risk of all-cause, CVD, and cancer mortality, considering participants not using antihypertensive medication and following an unfavorable lifestyle as the reference. In model 1, we adjusted for age (continuous) and sex (male vs female). In model 2, we further adjusted for education attainment (<high school, high school or equivalent, or ≥college), drinking status (current, former, or never drinker), hypertension duration (≤5, >5-10, or >10 years), self-reported physician-diagnosed diabetes or CVD, and uses of hypoglycemic and lipid-lowering medication. In model 3, we additionally adjusted for systolic and diastolic BP, fasting glucose, and HDL cholesterol levels and estimated glomerular filtration rate (all continuous). These covariates were selected based on prior literature.^21,22^ The proportional hazard assumption was tested based on Schoenfeld residuals and no violation was found. The generalized linear model was used to investigate the associations of overall lifestyle with blood glucose and lipids at baseline. A restricted cubic spline model with 3 knots (25th, 50th, and 75th) was applied to test dose-response associations between lifestyle score and mortality among participants with hypertension with and without antihypertensive medication use.

#### Changes in Lifestyle and Risk of Mortality

To evaluate the association between changes in lifestyle and risk of mortality, we conducted analysis in a subgroup of 6863 participants who completed both baseline and the first follow-up surveys. Changes in lifestyle were assessed from baseline to the first follow-up, and deaths were identified after the first follow-up. Lifestyle score greater than 5 was considered as high; less than 5, low. Participants were categorized into 4 groups: consistently low, high to low, low to high, and consistently high. The covariates adjusted in the multivariable model were obtained in the first follow-up, with the consistently low group as the reference.

#### Secondary Analysis

Stratified analyses were conducted by age (<65 vs ≥65 years), sex (male vs female), education attainment (<high school, high school, or ≥college), duration of hypertension (≤10 vs >10 years), and history of diabetes (yes vs no). In addition, several sensitivity and secondary analyses were performed. First, analyses were performed separately in participants using antihypertensive medication. Lifestyle score was included in models as categorical and continuous variables. Interaction associations between antihypertensive medication use (yes or no) and lifestyle (favorable, intermediate, or unfavorable) and mortality were tested using the likelihood ratio test by including an additional product term in the model. Second, we evaluated the associations of different components of lifestyle behaviors with all-cause mortality. Third, we also constructed a weighted lifestyle score based on β coefficients of each lifestyle factor in the Cox proportional hazards regression model with all 5 lifestyle factors included. Fourth, to further test the contribution of individual lifestyle factors, we omitted 1 lifestyle factor each time to reconstruct a new 8-point lifestyle score, and participants were categorized into scores 0 to 4, 5 to 6, and 7 to 8. Fifth, we excluded participants diagnosed with CVD before baseline or those died within 2 years of follow-up to minimize the potential reverse causation bias. Sixth, the main analysis was repeated only in participants with complete data. Finally, given the potential confounding of psychological factors in the association of interest,^23^ mental stress was further adjusted for in a subset of study population.

Analyses were performed using Stata statistical software, version MP 16.0 (StataCorp) and R version 3.6.1 (R Project for Statistical Computing). Two-tailed *P* < .05 was considered statistically significant. Data were analyzed from February to April 2021.

## Results

Table 1 shows the baseline characteristics of 14 392 participants with hypertension (mean [SD] age, 65.6 [7.4] years; 7277 [50.6%] men and 7115 [49.4%] women). Compared with participants not using antihypertensive medication and following an unfavorable lifestyle, those using antihypertensive medication and adhering to a favorable lifestyle had longer duration of hypertension (>10 years: 74 participants [33.5%] vs 2405 participants [53.7%]), higher prevalence of diabetes (51 participants [21.6%] vs 1285 participants [27.5%]) and CVD (53 participants [22.5%] vs 1550 participants [33.1%]), lower mean (SD) systolic (148 [22] mm Hg vs 142 [20] mm Hg) and diastolic (86 [13] mm Hg vs 81 [12] mm Hg) BP, and were more likely to use hypoglycemic (18 participants [7.6%] vs 807 participants [17.2%]) and lipid-lowing (22 participants [9.3%] vs 1370 participants [29.3%]) medications (Table 1). In addition, higher lifestyle score was associated with lower level of triglyceride and higher level of HDL cholesterol, regardless of antihypertensive medication use. Among patients using antihypertensive medication, higher lifestyle score was also associated with lower levels of fasting glucose and glycated hemoglobin A_1c_ (eTable 1 in the Supplement). There were differences in some characteristics of participants included and excluded from our analyses (eTable 2 in the Supplement).

**Table 1.** Baseline Characteristics According to Antihypertensive Medication Use and Lifestyle Score

Characteristic	No. (%)					
	Not using antihypertensive medication			Using antihypertensive medication		
	Score of 0-4	Score of 5-7	Score of 8-10	Score of 0-4	Score of 5-7	Score of 8-10
No.	236	1938	1594	637	5305	4682
Age, mean (SD), y	65.8 (7.3)	65.0 (7.3)	65.0 (7.8)	65.7 (7.4)	65.8 (7.2)	65.7 (7.6)
Sex						
Women	51 (21.6)	794 (41.0)	995 (62.4)	135 (21.2)	2235 (42.1)	2905 (62.0)
Men	185 (78.4)	1144 (59.0)	599 (37.6)	502 (78.8)	3070 (57.9)	1777 (38.0)
Education attainment						
<High school	178 (76.4)	1382 (71.9)	1063 (66.9)	437 (69.0)	3553 (67.4)	2884 (62.0)
High school or equivalent	40 (17.2)	378 (19.7)	397 (25.0)	134 (21.2)	1189 (22.6)	1173 (25.2)
≥College	15 (6.4)	162 (8.4)	129 (8.1)	62 (9.8)	527 (10.0)	597 (12.8)
Alcohol consumption						
Nondrinker	109 (46.2)	1154 (59.7)	1219 (76.5)	292 (45.8)	3434 (64.8)	3798 (81.2)
Current	110 (46.6)	642 (33.2)	307 (19.3)	267 (41.9)	1366 (25.8)	618 (13.2)
Former	17 (7.2)	137 (7.1)	67 (4.2)	78 (12.2)	500 (9.4)	262 (5.6)
Duration of hypertension, y						
≤5	103 (46.6)	1060 (56.9)	933 (60.2)	148 (24.8)	1270 (25.2)	1132 (25.3)
>5-10	44 (19.9)	308 (16.5)	220 (14.2)	135 (22.7)	1119 (22.2)	945 (21.1)
>10	74 (33.5)	495 (26.6)	397 (25.6)	313 (52.5)	2655 (52.6)	2405 (53.7)
Self-reported						
Diabetes	51 (21.6)	501 (25.9)	333 (20.9)	203 (31.9)	1653 (31.2)	1285 (27.5)
CVD	53 (22.5)	432 (22.3)	303 (19.0)	254 (39.9)	1884 (35.5)	1550 (33.1)
Use of hypoglycemic medication	18 (7.6)	181 (9.3)	145 (9.1)	124 (19.5)	1010 (19.0)	807 (17.2)
Use of lipid-lowering medication	22 (9.3)	199 (10.3)	172 (10.8)	199 (31.2)	1638 (30.9)	1370 (29.3)
Blood pressure, mm Hg						
Systolic	148 (22)	149 (20)	150 (21)	140 (20)	142 (20)	142 (20)
Diastolic	86 (13)	86 (12)	85 (12)	82 (12)	82 (12)	81 (12)
Fasting glucose, mean (SD), mg/dL	110.63 (39.28)	112.79 (36.58)	110.09 (29.73)	116.58 (39.28)	114.95 (34.77)	111.89 (32.25)
HDL cholesterol, mean (SD), mg/dL	54.44 (17.76)	55.60 (17.37)	57.53 (15.44)	52.9 (16.99)	53.67 (16.22)	55.98 (16.60)
eGFR, mean (SD), mL/min/1.73 m^2^	82.0 (33.9)	81.7 (24.5)	80.0 (23.5)	80.9 (36.4)	78.2 (29.4)	77.5 (24.5)

Abbreviations: CVD, cardiovascular disease; eGFR, estimated glomerular filtration rate; HDL, high-density lipoprotein.

SI conversion factors: To convert glucose to millimoles per liter, multiply by 0.0555; HDL cholesterol to millimoles per liter, multiply by 0.0259.

During a median (IQR) follow-up of 7.3 (5.7-10.3) years, we documented 2015 deaths, including 761 CVD deaths and 525 cancer deaths. Compared with participants not using antihypertensive medication and following an unfavorable lifestyle, those who used antihypertensive medications and had a favorable lifestyle had the lowest risk of all-cause mortality (hazard ratio [HR], 0.32; 95% CI, 0.25-0.42), CVD mortality (HR, 0.33; 95% CI, 0.21-0.53), and cancer mortality (HR, 0.30; 95% CI, 0.19-0.47) (Table 2). Furthermore, compared with the reference group, participants without antihypertensive medication treatment but who adopted a favorable lifestyle had lower risks of all-cause mortality (HR, 0.34; 95% CI, 0.25-0.46), CVD mortality (HR, 0.40; 95% CI, 0.24-0.67), and cancer mortality (HR, 0.33; 95% CI, 0.19-0.55); whereas those using antihypertensive medication but following an unfavorable lifestyle had no significant reduction in risk of CVD mortality (HR, 0.71; 95% CI, 0.43-1.17) or cancer mortality (HR, 0.64; 95% CI, 0.38-1.07).

**Table 2.** All-Cause and Cause-Specific Mortality According to Antihypertensive Medication Use and Lifestyle Score Among Participants With Hypertension

	HR (95% CI)					
	Not using antihypertensive medication, lifestyle score, points			Using antihypertensive medication, lifestyle score, points		
	0-4	5-7	8-10	0-4	5-7	8-10
Person-years, No.	1606	13 764	11 437	4694	41 943	38 142
Participants, No.	236	1938	1594	637	5305	4682
All-cause mortality						
Deaths, No.	63	243	138	163	855	553
Model 1^a^	1 [Reference]	0.49 (0.37-0.64)	0.34 (0.25-0.45)	0.80 (0.60-1.08)	0.48 (0.37-0.62)	0.35 (0.27-0.45)
Model 2^b^	1 [Reference]	0.47 (0.36-0.62)	0.34 (0.25-0.46)	0.70 (0.52-0.93)	0.43 (0.33-0.56)	0.32 (0.25-0.42)
Model 3^c^	1 [Reference]	0.48 (0.36-0.63)	0.34 (0.25-0.46)	0.70 (0.52- 0.94)	0.43 (0.33-0.56)	0.32 (0.25-0.42)
CVD mortality						
Deaths, No.	21	87	56	60	329	208
Model 1^a^	1 [Reference]	0.52 (0.32-0.84)	0.40 (0.24-0.66)	0.83 (0.51-1.37)	0.52 (0.34-0.82)	0.32 (0.25-0.42)
Model 2^b^	1 [Reference]	0.39 (0.23-0.64)	0.39 (0.23-0.64)	0.67 (0.40-1.10)	0.43 (0.28-0.68)	0.31 (0.20-0.49)
Model 3^c^	1 [Reference]	0.51 (0.32-0.83)	0.40 (0.24-0.67)	0.71 (0.43-1.17)	0.46 (0.29-0.72)	0.33 (0.21-0.53)
Cancer mortality						
Deaths, No.	22	60	43	44	206	150
Model 1^a^	1 [Reference]	0.34 (0.21-0.56)	0.30 (0.18-0.50)	0.65 (0.39-1.09)	0.34 (0.22-0.53)	0.28 (0.18-0.44)
Model 2^b^	1 [Reference]	0.35 (0.21-0.57)	0.32 (0.19-0.54)	0.64 (0.38-1.07)	0.35 (0.22-0.54)	0.30 (0.19-0.47)
Model 3^c^	1 [Reference]	0.35 (0.22-0.58)	0.33 (0.19-0.55)	0.64 (0.38-1.07)	0.35 (0.22-0.54)	0.30 (0.19-0.47)

Abbreviations: CVD, cardiovascular disease; HR, hazard ratio.

^a^ Adjusted for age (continuous), and sex (male vs female).

^b^ Further adjusted for education attainment (<high school, high school or equivalent, ≥college), drinking status (never drinker, former drinker, or current drinker), hypertension duration (≤5, >5 to 10, or >10 years), self-reported physician-diagnosed CVD (yes or no) and diabetes (yes or no), and uses of hypoglycemic (yes or no) or lipid-lowering (yes or no) medication.

^c^ Further adjusted for systolic blood pressure (continuous), diastolic blood pressure (continuous), fasting glucose (continuous), HDL cholesterol (continuous), and eGFR (continuous).

Dose-response analyses showed inverse linear associations between lifestyle score and all-cause, CVD, and cancer mortality, regardless of antihypertensive medication use (Figure). Each 1-point increase in lifestyle score was associated with a 17% lower risk of all-cause mortality, 15% lower risk of CVD mortality, and 18% lower risk of cancer mortality for participants not using antihypertensive medication, and a 14% lower risk of all-cause mortality, 14% lower risk of CVD mortality, and 13% lower risk of cancer mortality for those using medication. No significant interactions were found between antihypertensive medication use and lifestyle score (eTable 3 in the Supplement). For different combinations of lifestyle factors, the multivariable-adjusted model found a significant association per 1-point increase in lifestyle score when only BMI, smoking status, and diet were included in lifestyle score (adjusted HR [aHR], 0.92; 95% CI, 0.89-0.95). The risk decreased when physical activity and sleep duration were additionally included in the score (physical activity: aHR, 0.88; 95% CI, 0.85-0.90; sleep duration: aHR, 0.86; 95% CI, 0.83-0.88) (eTable 4 in the Supplement). Furthermore, the inverse associations between lifestyle score and risk of mortality were largely similar when using weighted lifestyle score (eTable 5 in the Supplement) or omitting 1 lifestyle factor each time from the total score (eTable 6 in the Supplement).

**Figure.**
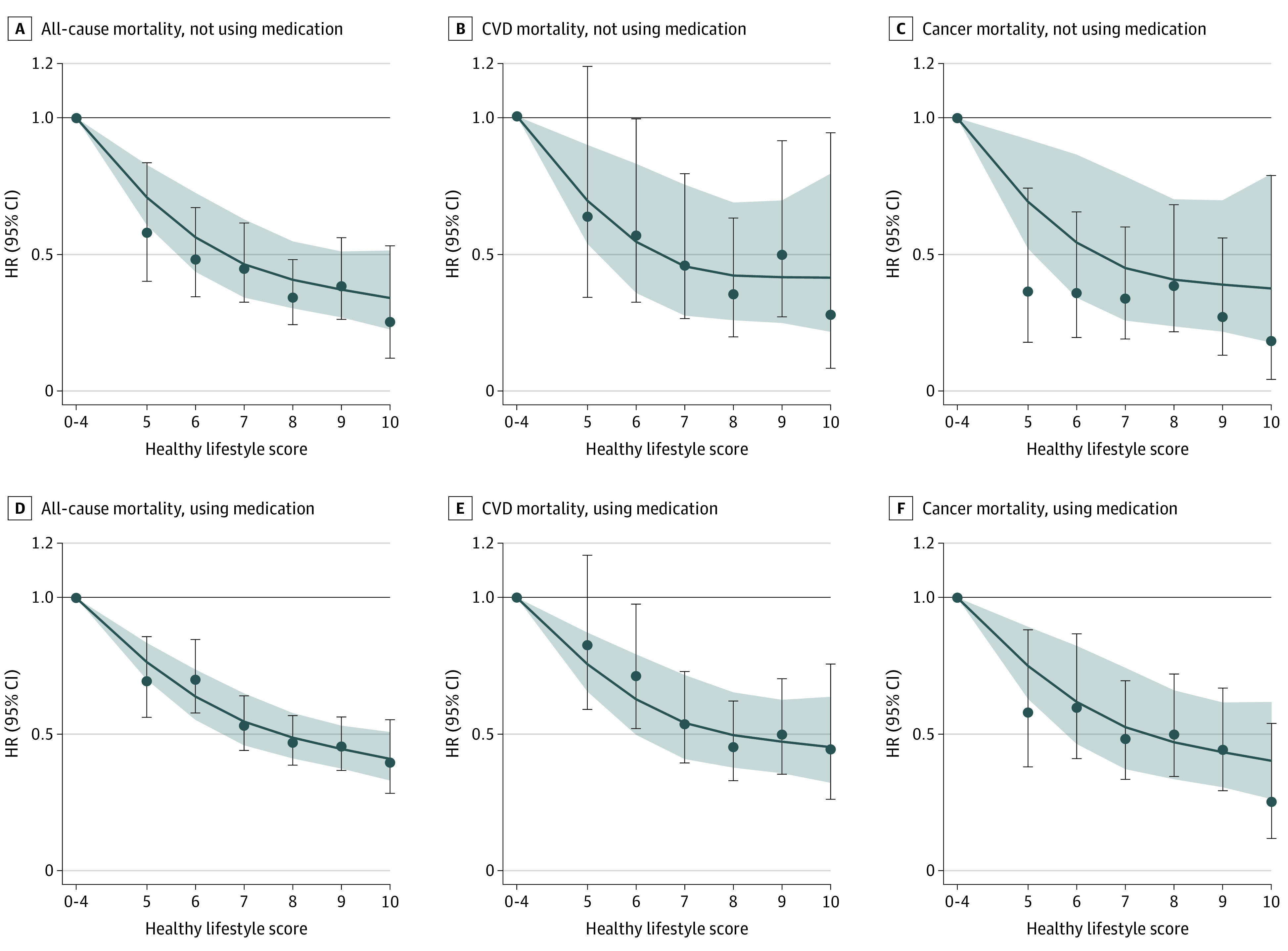
Dose-Response Association of Lifestyle Score With Mortality According to Hypertension Medication Use Lines indicate hazard ratios (HRs) estimated by restricted cubic spline model, considering reference as the mean lifestyle score among participants with 0-4 points; and shading, 95% CIs of estimated HRs.

Improvement in lifestyle after hypertension diagnosis was also significantly associated with lower risk of all-cause and cause-specific mortality (Table 3). Compared with participants with consistently low lifestyle score between baseline and first follow-up survey, participants who improved their lifestyle score (low to high) had decreased risk of all-cause mortality (HR, 0.52; 95% CI, 0.36-0.76), CVD mortality (HR, 0.53; 95% CI, 0.30-0.94), and cancer mortality (0.73; 95% CI, 0.37-1.44), and those having a consistently high lifestyle score also had reduced risk of all-cause mortality (HR, 0.51; 95% CI, 0.39-0.67), CVD mortality (HR, 0.51; 95% CI, 0.34-0.77), and cancer mortality (HR, 0.52; 95% CI, 0.30-0.89).

**Table 3.** All-Cause and Cause-Specific Mortality According to Changes in Lifestyle Score (2008-2013) Among Participants With Hypertension

Measure	Change in lifestyle score, HR (95% CI)			
	Consistently low	High to low	Low to high	Consistently high
Person-years, No.	2032	3236	3271	29 574
Participants, No.	308	607	595	5353
All-cause mortality				
Deaths, No.	65	100	49	409
Model 1^a^	1 [Reference]	0.92 (0.67-1.26)	0.51 (0.35-0.74)	0.47 (0.36-0.62)
Model 2^b^	1 [Reference]	0.95 (0.69-1.31)	0.53 (0.37-0.78)	0.52 (0.39-0.68)
Model 3^c^	1 [Reference]	0.94 (0.68-1.29)	0.52 (0.36-0.76)	0.51 (0.39-0.67)
CVD mortality				
Deaths, No.	29	49	21	170
Model 1^a^	1 [Reference]	1.03 (0.65-1.65)	0.50 (0.28-0.88)	0.46 (0.31-0.69)
Model 2^b^	1 [Reference]	1.08 (0.68-1.73)	0.54 (0.31-0.96)	0.53 (0.35-0.79)
Model 3^c^	1 [Reference]	1.06 (0.66-1.70)	0.53 (0.30-0.94)	0.51 (0.34-0.77)
Cancer mortality				
Deaths, No.	16	24	18	107
Model 1^a^	1 [Reference]	0.88 (0.47-1.67)	0.71 (0.36-1.39)	0.48 (0.28-0.82)
Model 2^b^	1 [Reference]	0.93 (0.49-1.75)	0.73 (0.37-1.44)	0.51 (0.30-0.86)
Model 3^c^	1 [Reference]	0.94 (0.50-1.79)	0.73 (0.37-1.44)	0.52 (0.30-0.89)

Abbreviations: CVD, cardiovascular disease; HR, hazard ratio.

^a^ Adjusted for age (continuous), and sex (male vs female).

^b^ Further adjusted for education attainment (<high school, high school or equivalent, ≥college), drinking status (never drinker, former drinker, or current drinker), hypertension duration (≤5, >5 to 10, or >10 years), self-reported physician-diagnosed CVD (yes or no) and diabetes (yes or no), and uses of hypoglycemic (yes or no) or lipid-lowering (yes or no) medication.

^c^ Further adjusted for systolic blood pressure (continuous), diastolic blood pressure (continuous), fasting glucose (continuous), HDL cholesterol (continuous), and eGFR (continuous).

Consistent results were observed in subgroup analyses stratified by age, sex, education attainment, duration of hypertension, and self-reported diabetes (eFigure 2 in the Supplement). The results were essentially unchanged when excluding participants diagnosed with CVD before baseline (eTable 7 in the Supplement), excluding participants who died within 2 years of follow-up (eTable 8 in the Supplement), or only including participants with complete data (eTable 9 in the Supplement). The results were attenuated with additional adjustment for mental stress (eTable 10 in the Supplement). Similar findings were observed when using the Asian BMI cutoff (eTable 11 in the Supplement).

## Discussion

Among participants with hypertension in a prospective cohort in China, we found that the combination of using antihypertensive medication and adhering to a favorable lifestyle (weight within reference range, nonsmoking, adequate physical activity, high-quality diet, and optimal sleep duration) was significantly associated with lower risks of mortality. The association was independent of traditional risk factors, including hypertension duration, common comorbidities, use of hypoglycemic and lipid-lowering medication, metabolic biomarkers, and mental stress. In addition, improvement in lifestyle after hypertension diagnosis was also associated with lower risk of subsequent premature death. The stratified and sensitivity analyses demonstrated the robustness of the findings.

Large-scale population-based studies have reported that nonadherence to or discontinuation of antihypertensive medication was associated with higher risks of stroke or myocardial infarction.^8,24,25^ However, the adherence to medication treatment decreased over time. An Italian study of 13 303 patients with newly diagnosed hypertension reported that 42.6% of participants stopped any antihypertensive medication within the first year after hypertension diagnosis.^26^ Through linking to drug-dispensing records from community pharmacies and hospital discharge records, a study in the Netherlands identified 2325 participants aged 40 to 59 years who initiated antihypertensive medication. They found that 22% of participants temporarily stopped and restarted using medication and only 39% of participants used the medication continuously during 10 years of follow-up.^27^ According to a systematic analysis of 968 419 adults from 90 countries, there were large global disparities in hypertension treatment and control.^28^

Adherence to antihypertensive medication is certainly an effective strategy to lower BP and prevent cardiovascular events.^29^ The benefit would be greater along with lifestyle modification, which has been highlighted as the first-line treatment of hypertension in 2020 International Society of Hypertension Global Hypertension Practice Guidelines.^7^ The UK Biobank study found that maintaining healthy lifestyle factors (BMI, diet, smoking, alcohol consumption, sodium excretion, and sedentary behavior) was associated with 3.5 mm Hg lower systolic BP and approximately 30% lower risk of CVD, regardless of genetic susceptibility to hypertension.^30^ In addition, several randomized clinical trials among participants with nonoptimal BP or hypertension supported the beneficial effects of lifestyle modification on BP control and cardiovascular health. For example, in the PREMIER Clinical Trial of 810 participants with nonoptimal BP, lifestyle intervention through weight loss, sodium restriction, enhanced physical activity, limited alcohol intake, and improving dietary quality over 6 months significantly reduced systolic BP (by 4.3 mm Hg) and the risk of coronary heart disease (by 12%).^21,22^ In the Chinese Hypertension Intervention Efficacy study, including 12 245 participants with hypertension, conducting a lifestyle intervention using health education over 3.5 years, found that participants with improvements in at least 2 lifestyle factors, including weight loss, increasing exercise, and healthy diet, had a 55% lower risk of cardiovascular events.^31^

Evidence regarding the joint association of antihypertensive medication and lifestyle with cardiovascular health is limited and somewhat mixed. A 2020 trial by Xiao et al^32^ found that the combination of lifestyle and medication intervention led to better BP control than medication alone. However, 2 cross-sectional studies reported no significant association between lifestyle and BP control for participants who were using antihypertensive medication.^33,34^ In addition, 2 observational studies in Finland reported that participants with hypertension who were not using antihypertensive medication but adopted at least 3 healthy behaviors (never smoking, weight within reference range, moderate or vigorous exercise, vegetable consumption ≥3 times/week, and limited alcohol intake) had significantly lower risk of stroke or heart failure compared with those using antihypertensive medication but following fewer than 3 healthy behaviors.^14,15^ Notably, these studies were mostly performed among Western populations, and some studies were limited by small sample size, cross-sectional study design, simple and noncomprehensive assessment on overall lifestyle, no information on changes in lifestyle, and insufficient adjustment for several important covariates, such as duration of hypertension and comorbidity.

Additionally, to our knowledge, no study has examined the combined association of antihypertensive medication treatment and lifestyle with mortality risk among participants with hypertension. In this study, we found that individuals using antihypertensive medication and following a favorable lifestyle had the lowest risk of all-cause, CVD, and cancer mortality. There were inverse linear associations between lifestyle score and mortality, regardless of hypertensive medication use. Additionally, for participants with an unfavorable lifestyle, there was no significant reduction in mortality risk even if they were using antihypertensive medication. Furthermore, we found that improvement in lifestyle after hypertension diagnosis was associated with significantly lower risk of subsequent death. From a public health perspective, BP management and complication prevention have great health and socioeconomic benefits, considering that the annual cost of hypertension has reached tens of billions of dollars per country.^35^ Adherence to an antihypertensive medication regimen is no doubt an efficient way to control BP; however, it is not advisable to only rely on medication and ignore the role of lifestyle for the prevention of poor prognosis. Our findings further support that the combination of antihypertensive medication treatment and adopting a healthier lifestyle (even for those with an unfavorable lifestyle at hypertension diagnosis but who improve it later) could maximize the health benefits.

To our knowledge, we are among the first to investigate the combinations of antihypertensive medication use and lifestyle, as well as changes in lifestyle, in association with risk of all-cause, CVD, and cancer mortality. The underlying mechanisms for the beneficial associations of these lifestyle behaviors may involve various pathways. Each behavior might have an impact on BP by modulating visceral fat accumulation, insulin resistance, rennin-angiotensin-aldosterone system, vascular endothelial function, oxidative stress, inflammation, and/or autonomic function.^12^ A 10–mm Hg reduction in systolic BP or a 5–mm Hg reduction in diastolic BP was associated with 10% to 30% lower risk of major CVD events and 11% to 13% lower risk of all-cause mortality.^36,37^ Besides the associations with BP, these lifestyle factors may have associations with CVD and deaths through a series of metabolic and molecular alterations manifested as inhibiting insulin resistance, inflammation, and oxidative stress and slowing the accumulation of cellular and organ damage.^38,39,40,41,42^

### Limitations

This study has some limitations. First, we asked about hypertensive medication use within the last 2 weeks, which may not represent long-term medication adherence or whether the participants used the prescription on time. However, 74% of participants with hypertension had the same answer to antihypertensive medication use at baseline and first follow-up. In addition, there was no information on the number and dosage of antihypertensive medication; thus, we could not comprehensively evaluate the direct association of medication with blood pressure, which would require detailed investigation in future studies. Second, we believe our findings are generalizable to middle-aged and older Asian populations, but they would need to be replicated in other ethnicities and age groups. Third, the 5 factors included in the lifestyle score might not represent all aspects of lifestyle, although they were the major modifiable components and mainly reported in previous literatures. Additionally, we adjusted for comprehensive covariates, and the results remained consistent. Fourth, diet was assessed using a simple food frequency questionnaire without information on portions size; hence, we were unable to assess total energy intake in the model. Nevertheless, the association of vegetable, fruit, and meat intake frequency with CVD risk was reported in another large prospective study of Chinese population.^43^ Fifth, measurement errors were inevitable in self-reported assessments of lifestyle factors. However, owing to the prospective study design, the misclassifications would likely be nondifferential and tended to attenuate the observed association to null. Sixth, selection bias could be possible owing to the differences in baseline demographics, lifestyles, and comorbidities between participants included and excluded from this analysis. Seventh, owing to the observational study design, the residual confounding could not be completely ruled out.

## Conclusions

This cohort study found that the combination of using antihypertensive medication and adhering to a favorable lifestyle was associated with significantly lower risk of all-cause, CVD, and cancer mortality. In addition, improvement in lifestyle after hypertension diagnosis was also associated with lower risk of mortality. Our findings highlight that, in addition to using antihypertensive medication, following a favorable lifestyle was associated with benefits in preventing or delaying premature death among individuals with hypertension. For the management of hypertension, advocating regular medication is important but not sufficient; long-term adherence to a favorable lifestyle may yield greater benefits.

## Notes

Supplement. **eFigure 1.** Timeline of the DFTJ Cohort and Flowchart of Study Participants **eFigure 2.** Stratified Analysis of the Association of Antihypertensive Medication Use and Lifestyle Score With All-Cause Mortality **eTable 1.** Least-Square Means of Metabolic Biomarkers According to Lifestyle Score Stratified by Antihypertensive Medication Use **eTable 2.** Baseline Characteristics for Participants Included and Excluded **eTable 3.** HRs (95% CIs) of All-Cause and Cause-Specific Mortality According to Lifestyle Score Stratified by Antihypertensive Medication Use **eTable 4.** HRs (95% CIs) for 1-Point Increase of Lifestyle Score According to Different Combination of Lifestyle Factors **eTable 5.** Association of Unweighted and Weighted Lifestyle Score With the Risk of All-Cause, CVD, and Cancer Mortality **eTable 6. **Associations of Different Lifestyle Score Consisting of 4 Lifestyle Factors with Risks of Mortality **eTable 7.** HRs (95% CIs) for All-Cause and Cause-Specific Mortality by Antihypertensive Medication Use and Lifestyle Score After Excluding Participants With CVD at Baseline **eTable 8.** HRs (95% CIs) for All-Cause and Cause-Specific Mortality by Antihypertensive Medication Use and Lifestyle Score After Excluding Participants Died Within 2 Years of Follow-up **eTable 9.** HRs (95% CIs) for All-Cause and Cause-Specific Mortality by Antihypertensive Medication Use and Lifestyle Score Among Participants With Complete Data **eTable 10.** HRs (95% CIs) for All-Cause and Cause-Specific Mortality by Antihypertensive Medication Use and Lifestyle Score With Further Adjustment for Mental Stress **eTable 11.** HRs (95% CIs) for All-Cause and Cause-Specific Mortality by Antihypertensive Medication Use and Lifestyle Score When Using Asian BMI Cutoff

## References

[1] Collaboration NCDRF; NCD Risk Factor Collaboration (NCD-RisC). Worldwide trends in blood pressure from 1975 to 2015: a pooled analysis of 1479 population-based measurement studies with 19.1 million participants. Lancet. 2017;389(10064):37-55. doi:10.1016/S0140-6736(16)31919-5 27863813 PMC5220163

[2] Mills KT, Stefanescu A, He J. The global epidemiology of hypertension. Nat Rev Nephrol. 2020;16(4):223-237. doi:10.1038/s41581-019-0244-2 32024986 PMC7998524

[3] Bundy JD, Li C, Stuchlik P, . Systolic blood pressure reduction and risk of cardiovascular disease and mortality: a systematic review and network meta-analysis. JAMA Cardiol. 2017;2(7):775-781. doi:10.1001/jamacardio.2017.1421 28564682 PMC5710614

[4] GBD 2019 Risk Factors Collaborators. Global burden of 87 risk factors in 204 countries and territories, 1990-2019: a systematic analysis for the Global Burden of Disease Study 2019. Lancet. 2020;396(10258):1223-1249. doi:10.1016/S0140-6736(20)30752-2 33069327 PMC7566194

[5] Whelton PK, Carey RM, Aronow WS, . 2017 ACC/AHA/AAPA/ABC/ACPM/AGS/APhA/ASH/ASPC/NMA/PCNA guideline for the prevention, detection, evaluation, and management of high blood pressure in adults: a report of the American College of Cardiology/American Heart Association Task Force on Clinical Practice Guidelines. J Am Coll Cardiol. 2018;71(19):e127-e248. doi:10.1016/j.jacc.2017.11.006 29146535

[6] Chow CK, Teo KK, Rangarajan S, ; PURE (Prospective Urban Rural Epidemiology) Study investigators. Prevalence, awareness, treatment, and control of hypertension in rural and urban communities in high-, middle-, and low-income countries. JAMA. 2013;310(9):959-968. doi:10.1001/jama.2013.184182 24002282

[7] Unger T, Borghi C, Charchar F, . 2020 International Society of Hypertension global hypertension practice guidelines. Hypertension. 2020;75(6):1334-1357. doi:10.1161/HYPERTENSIONAHA.120.15026 32370572

[8] Herttua K, Martikainen P, Batty GD, Kivimäki M. Poor adherence to statin and antihypertensive therapies as risk factors for fatal stroke. J Am Coll Cardiol. 2016;67(13):1507-1515. doi:10.1016/j.jacc.2016.01.044 27150680 PMC4863178

[9] Kim S, Shin DW, Yun JM, . Medication adherence and the risk of cardiovascular mortality and hospitalization among patients with newly prescribed antihypertensive medications. Hypertension. 2016;67(3):506-512. doi:10.1161/HYPERTENSIONAHA.115.06731 26865198

[10] Li Y, Schoufour J, Wang DD, . Healthy lifestyle and life expectancy free of cancer, cardiovascular disease, and type 2 diabetes: prospective cohort study. BMJ. 2020;368:l6669. doi:10.1136/bmj.l6669 31915124 PMC7190036

[11] Li Y, Pan A, Wang DD, . Impact of healthy lifestyle factors on life expectancies in the US population. Circulation. 2018;138(4):345-355. doi:10.1161/CIRCULATIONAHA.117.032047 29712712 PMC6207481

[12] Valenzuela PL, Carrera-Bastos P, Galvez BG, . Lifestyle interventions for the prevention and treatment of hypertension. Nat Rev Cardiol. 2021;18(4):251-275. doi:10.1038/s41569-020-00437-9 33037326

[13] Cook R, Lamont T, Martin R; NIHR Dissemination Centre. Lifestyle changes may be more important than drugs for mild hypertension. BMJ. 2019;364:l571. doi:10.1136/bmj.l571 30770340

[14] Wang Y, Tuomilehto J, Jousilahti P, . Healthy lifestyle status, antihypertensive treatment and the risk of heart failure among Finnish men and women. J Hypertens. 2013;31(11):2158-2164. doi:10.1097/HJH.0b013e328364136d 23846864

[15] Zhang Y, Tuomilehto J, Jousilahti P, Wang Y, Antikainen R, Hu G. Lifestyle factors and antihypertensive treatment on the risks of ischemic and hemorrhagic stroke. Hypertension. 2012;60(4):906-912. doi:10.1161/HYPERTENSIONAHA.112.193961 22868396

[16] Wang F, Zhu J, Yao P, . Cohort profile: the Dongfeng-Tongji cohort study of retired workers. Int J Epidemiol. 2013;42(3):731-740. doi:10.1093/ije/dys053 22531126

[17] Physical status: the use and interpretation of anthropometry: report of a WHO Expert Committee. World Health Organ Tech Rep Ser. 1995;854:1-452. 8594834

[18] Eckel RH, Jakicic JM, Ard JD, ; American College of Cardiology/American Heart Association Task Force on Practice Guidelines. 2013 AHA/ACC guideline on lifestyle management to reduce cardiovascular risk: a report of the American College of Cardiology/American Heart Association Task Force on Practice Guidelines. J Am Coll Cardiol. 2014;63(25 Pt B):2960-2984. doi:10.1016/j.jacc.2013.11.003 24239922

[19] Wang C, Bangdiwala SI, Rangarajan S, . Association of estimated sleep duration and naps with mortality and cardiovascular events: a study of 116 632 people from 21 countries. Eur Heart J. 2019;40(20):1620-1629. doi:10.1093/eurheartj/ehy695 30517670 PMC6528160

[20] Levey AS, Stevens LA, Schmid CH, ; CKD-EPI (Chronic Kidney Disease Epidemiology Collaboration). A new equation to estimate glomerular filtration rate. Ann Intern Med. 2009;150(9):604-612. doi:10.7326/0003-4819-150-9-200905050-00006 19414839 PMC2763564

[21] Appel LJ, Champagne CM, Harsha DW, ; Writing Group of the PREMIER Collaborative Research Group. Effects of comprehensive lifestyle modification on blood pressure control: main results of the PREMIER clinical trial. JAMA. 2003;289(16):2083-2093. 12709466 10.1001/jama.289.16.2083

[22] Maruthur NM, Wang NY, Appel LJ. Lifestyle interventions reduce coronary heart disease risk: results from the PREMIER Trial. Circulation. 2009;119(15):2026-2031. doi:10.1161/CIRCULATIONAHA.108.809491 19349322 PMC2995494

[23] Steptoe A, Kivimäki M. Stress and cardiovascular disease. Nat Rev Cardiol. 2012;9(6):360-370. doi:10.1038/nrcardio.2012.45 22473079

[24] Herttua K, Tabák AG, Martikainen P, Vahtera J, Kivimäki M. Adherence to antihypertensive therapy prior to the first presentation of stroke in hypertensive adults: population-based study. Eur Heart J. 2013;34(38):2933-2939. doi:10.1093/eurheartj/eht219 23861328 PMC3791393

[25] Breekveldt-Postma NS, Penning-van Beest FJ, Siiskonen SJ, . The effect of discontinuation of antihypertensives on the risk of acute myocardial infarction and stroke. Curr Med Res Opin. 2008;24(1):121-127. doi:10.1185/030079908X253843 18031596

[26] Mazzaglia G, Mantovani LG, Sturkenboom MC, . Patterns of persistence with antihypertensive medications in newly diagnosed hypertensive patients in Italy: a retrospective cohort study in primary care. J Hypertens. 2005;23(11):2093-2100. doi:10.1097/01.hjh.0000186832.41125.8a 16208153

[27] Van Wijk BL, Klungel OH, Heerdink ER, de Boer A. Rate and determinants of 10-year persistence with antihypertensive drugs. J Hypertens. 2005;23(11):2101-2107. doi:10.1097/01.hjh.0000187261.40190.2e 16208154

[28] Mills KT, Bundy JD, Kelly TN, . Global disparities of hypertension prevalence and control: a systematic analysis of population-based studies from 90 countries. Circulation. 2016;134(6):441-450. doi:10.1161/CIRCULATIONAHA.115.018912 27502908 PMC4979614

[29] Law MR, Morris JK, Wald NJ. Use of blood pressure lowering drugs in the prevention of cardiovascular disease: meta-analysis of 147 randomised trials in the context of expectations from prospective epidemiological studies. BMJ. 2009;338:b1665. doi:10.1136/bmj.b1665 19454737 PMC2684577

[30] Pazoki R, Dehghan A, Evangelou E, . Genetic predisposition to high blood pressure and lifestyle factors: associations with midlife blood pressure levels and cardiovascular events. Circulation. 2018;137(7):653-661. doi:10.1161/CIRCULATIONAHA.117.030898 29254930

[31] Hua K, Hao G, Li W. Cardiovascular outcomes of lifestyle intervention in hypertensive patients with antihypertensive agents. Int J Cardiol. 2017;227:751-756. doi:10.1016/j.ijcard.2016.10.062 27810294

[32] Xiao J, Ren WL, Liang YY, . Effectiveness of lifestyle and drug intervention on hypertensive patients: a randomized community intervention trial in rural China. J Gen Intern Med. 2020;35(12):3449-3457. doi:10.1007/s11606-019-05601-7 33021715 PMC7728841

[33] Akbarpour S, Khalili D, Zeraati H, Mansournia MA, Ramezankhani A, Fotouhi A. Healthy lifestyle behaviors and control of hypertension among adult hypertensive patients. Sci Rep. 2018;8(1):8508. doi:10.1038/s41598-018-26823-5 29855520 PMC5981436

[34] Kimani S, Mirie W, Chege M, Okube OT, Muniu S. Association of lifestyle modification and pharmacological adherence on blood pressure control among patients with hypertension at Kenyatta National Hospital, Kenya: a cross-sectional study. BMJ Open. 2019;9(1):e023995. doi:10.1136/bmjopen-2018-023995 30782721 PMC6340423

[35] Wierzejska E, Giernaś B, Lipiak A, Karasiewicz M, Cofta M, Staszewski R. A global perspective on the costs of hypertension: a systematic review. Arch Med Sci. 2020;16(5):1078-1091. doi:10.5114/aoms.2020.92689 32863997 PMC7444692

[36] Ettehad D, Emdin CA, Kiran A, . Blood pressure lowering for prevention of cardiovascular disease and death: a systematic review and meta-analysis. Lancet. 2016;387(10022):957-967. doi:10.1016/S0140-6736(15)01225-8 26724178

[37] Thomopoulos C, Parati G, Zanchetti A. Effects of blood pressure lowering on outcome incidence in hypertension—1: overview, meta-analyses, and meta-regression analyses of randomized trials. J Hypertens. 2014;32(12):2285-2295. doi:10.1097/HJH.0000000000000378 25255397

[38] Tobaldini E, Fiorelli EM, Solbiati M, Costantino G, Nobili L, Montano N. Short sleep duration and cardiometabolic risk: from pathophysiology to clinical evidence. Nat Rev Cardiol. 2019;16(4):213-224. doi:10.1038/s41569-018-0109-6 30410106

[39] Veronese N, Li Y, Manson JE, Willett WC, Fontana L, Hu FB. Combined associations of body weight and lifestyle factors with all cause and cause specific mortality in men and women: prospective cohort study. BMJ. 2016;355:i5855. doi:10.1136/bmj.i5855 27884868 PMC5122318

[40] Rizza W, Veronese N, Fontana L. What are the roles of calorie restriction and diet quality in promoting healthy longevity? Ageing Res Rev. 2014;13:38-45. doi:10.1016/j.arr.2013.11.002 24291541

[41] Booth FW, Roberts CK, Laye MJ. Lack of exercise is a major cause of chronic diseases. Compr Physiol. 2012;2(2):1143-1211. doi:10.1002/cphy.c110025 23798298 PMC4241367

[42] Ambrose JA, Barua RS. The pathophysiology of cigarette smoking and cardiovascular disease: an update. J Am Coll Cardiol. 2004;43(10):1731-1737. doi:10.1016/j.jacc.2003.12.047 15145091

[43] Lv J, Yu C, Guo Y, ; China Kadoorie Biobank Collaborative Group. Adherence to healthy lifestyle and cardiovascular diseases in the Chinese population. J Am Coll Cardiol. 2017;69(9):1116-1125. doi:10.1016/j.jacc.2016.11.076 28254173 PMC6675601

