# Supplementary material for: Association of Lifestyle Factors and Antihypertensive Medication Use With Risk of All-Cause and Cause-Specific Mortality Among Adults With Hypertension in China
Source: JAMA Netw Open. 2022 Feb 1;5(2):e2146118. doi: 10.1001/jamanetworkopen.2021.46118 (PMC8808332; doi:10.1001/jamanetworkopen.2021.46118)
Supplement: Supplement. — eFigure 1. Timeline of the DFTJ Cohort and Flowchart of Study Participants eFigure 2. Stratified Analysis of the Association of Antihypertensive Medication Use and Lifestyle Score With All-Cause Mortality eTable 1. Least-Square Means of Metabolic Biomarkers According to Lifestyle Score Stratified by Antihypertensive Medication Use eTable 2. Baseline Characteristics for Participants Included and Excluded eTable 3. HRs (95% CIs) of All-Cause and Cause-Specific Mortality According to Lifestyle Score Stratified by Antihypertensive Medication Use eTable 4. HRs (95% CIs) for 1-Point Increase of Lifestyle Score According to Different Combination of Lifestyle Factors eTable 5. Association of Unweighted and Weighted Lifestyle Score With the Risk of All-Cause, CVD, and Cancer Mortality eTable 6. Associations of Different Lifestyle Score Consisting of 4 Lifestyle Factors with Risks of Mortality eTable 7. HRs (95% CIs) for All-Cause and Cause-Specific Mortality by Antihypertensive Medication Use and Lifestyle Score After Excluding Participants With CVD at Baseline eTable 8. HRs (95% CIs) for All-Cause and Cause-Specific Mortality by Antihypertensive Medication Use and Lifestyle Score After Excluding Participants Died Within 2 Years of Follow-up eTable 9. HRs (95% CIs) for All-Cause and Cause-Specific Mortality by Antihypertensive Medication Use and Lifestyle Score Among Participants With Complete Data eTable 10. HRs (95% CIs) for All-Cause and Cause-Specific Mortality by Antihypertensive Medication Use and Lifestyle Score With Further Adjustment for Mental Stress eTable 11. HRs (95% CIs) for All-Cause and Cause-Specific Mortality by Antihypertensive Medication Use and Lifestyle Score When Using Asian BMI Cutoff [file jamanetwopen-e2146118-s001.pdf]

Supplemental Online Content

Lu Q, Zhang Y, Geng T, et al. Association of lifestyle factors and antihypertensive medication use with risk of all-cause and cause-specific mortality among adults with hypertension in China. *JAMA Netw Open*. 2022;5(2):e2146118. doi:10.1001/jamanetworkopen.2021.46118

eFigure 1. Timeline of the DFTJ Cohort and Flowchart of Study Participants
eFigure 2. Stratified Analysis of the Association of Antihypertensive Medication Use and Lifestyle Score With All-Cause Mortality
eTable 1. Least-Square Means of Metabolic Biomarkers According to Lifestyle Score Stratified by Antihypertensive Medication Use
eTable 2. Baseline Characteristics for Participants Included and Excluded
eTable 3. HRs (95% CIs) of All-Cause and Cause-Specific Mortality According to Lifestyle Score Stratified by Antihypertensive Medication Use
eTable 4. HRs (95% CIs) for 1-Point Increase of Lifestyle Score According to Different Combination of Lifestyle Factors
eTable 5. Association of Unweighted and Weighted Lifestyle Score With the Risk of All-Cause, CVD, and Cancer Mortality
eTable 6. Associations of Different Lifestyle Score Consisting of 4 Lifestyle Factors with Risks of Mortality
eTable 7. HRs (95% CIs) for All-Cause and Cause-Specific Mortality by Antihypertensive Medication Use and Lifestyle Score After Excluding Participants With CVD at Baseline
eTable 8. HRs (95% CIs) for All-Cause and Cause-Specific Mortality by Antihypertensive Medication Use and Lifestyle Score After Excluding Participants Died Within 2 Years of Follow-up
eTable 9. HRs (95% CIs) for All-Cause and Cause-Specific Mortality by Antihypertensive Medication Use and Lifestyle Score Among Participants With Complete Data
eTable 10. HRs (95% CIs) for All-Cause and Cause-Specific Mortality by Antihypertensive Medication Use and Lifestyle Score With Further Adjustment for Mental Stress
eTable 11. HRs (95% CIs) for All-Cause and Cause-Specific Mortality by Antihypertensive Medication Use and Lifestyle Score When Using Asian BMI Cutoff

This supplemental material has been provided by the authors to give readers additional information about their work.

© 2022 Lu Q et al. *JAMA Network Open.*

eFigure 1. Timeline of the DFTJ Cohort and Flowchart of Study Participants

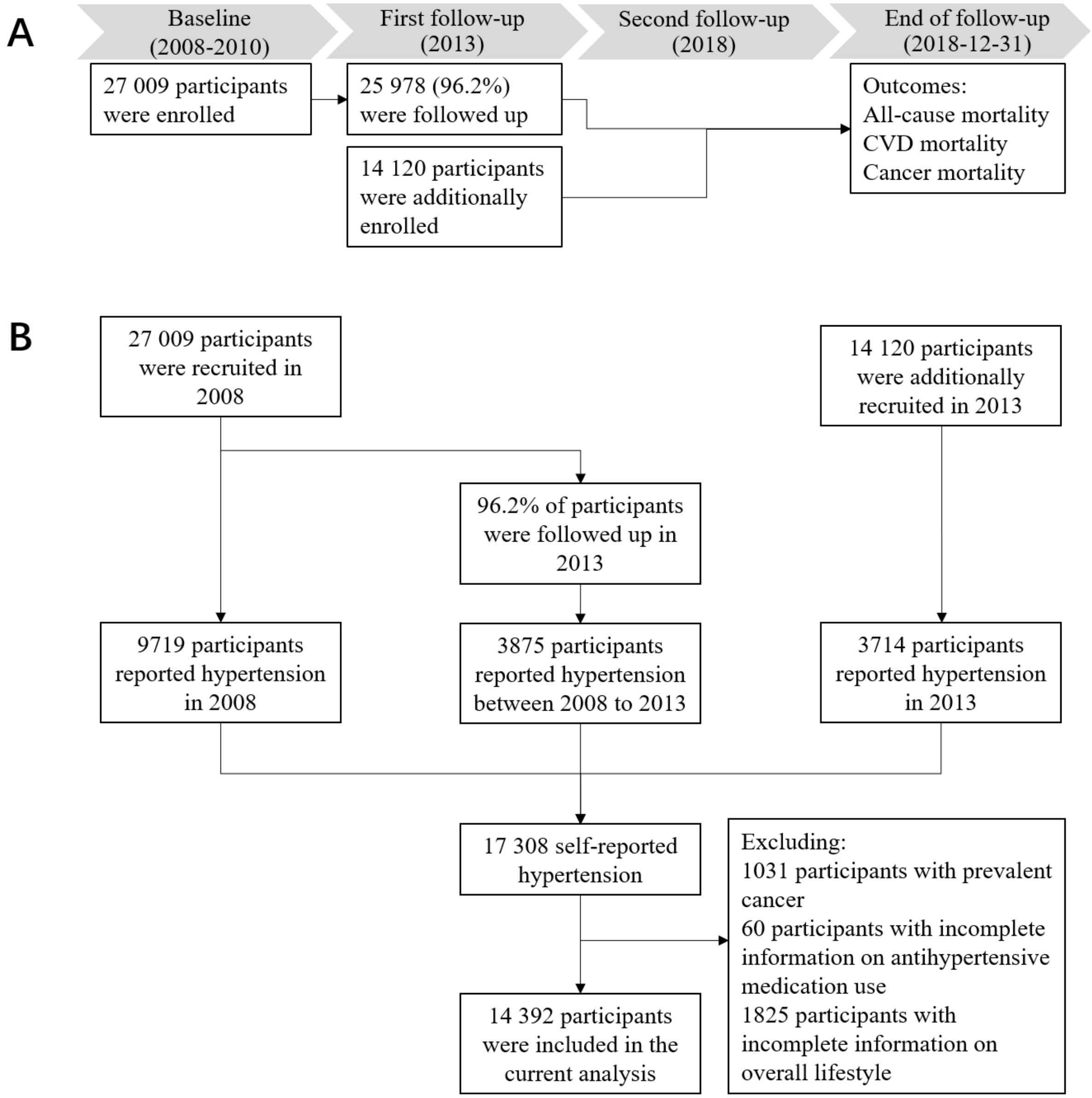

© 2022 Lu Q et al. *JAMA Network Open.*

eFigure 2. Stratified Analysis of the Association of Antihypertensive Medication Use and Lifestyle Score With All-Cause Mortality

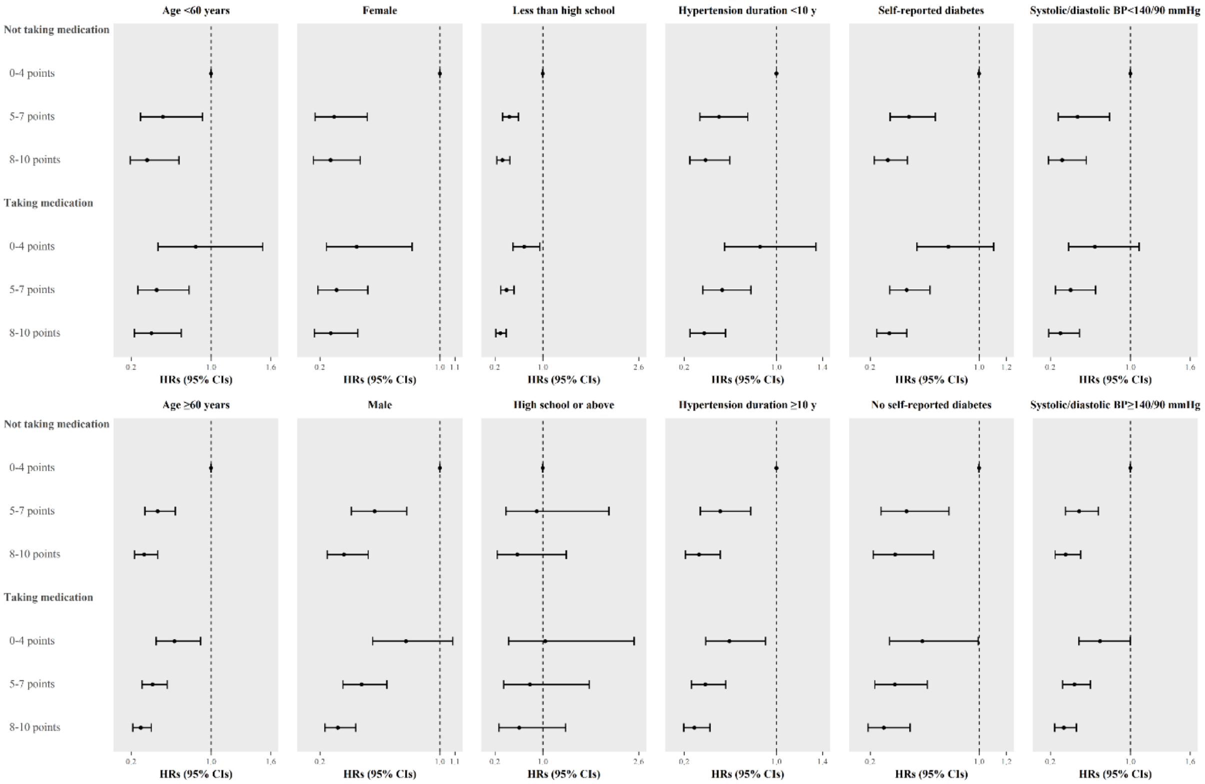

Adjusted for age (continuous), and sex (male, or female), education attainment (less than high school, high school or equivalent, or college or above), drinking status (never drinker, former drinker, or current drinker), hypertension duration (≤3, >3 to ≤10, or >10 years), self-reported physician-diagnosed CVD (yes, or no) and diabetes (yes, or no), uses of hypoglycemic (yes, or no) and lipid-lowing medication (yes, or no), systolic blood pressure (continuous), diastolic blood pressure (continuous), fasting glucose (continuous), HDL cholesterol (continuous), and eGFR (continuous). Stratified factors were not adjusted in each model.

© 2022 Lu Q et al. *JAMA Network Open.*

eTable 1. Least-Square Means of Metabolic Biomarkers According to Lifestyle Score Stratified by Antihypertensive Medication Use*

	Overall lifestyle score			*P* trend
	0-4 points	5-7 points	8-10 points	
**Not taking antihypertensive medication**				
Fasting glucose (n=3648), mmol/L	6.84 (0.22)	6.86 (0.19)	6.84 (0.20)	0.80
HbA1c (n=1949), %	6.56 (0.20)	6.50 (0.18)	6.44 (0.18)	0.13
Total cholesterol (n=3562), mmol/L	4.97 (0.14)	5.02 (0.13)	5.02 (0.13)	0.53
HDL cholesterol (n=3659), mmol/L	1.31 (0.06)	1.33 (0.05)	1.37 (0.05)	0.008
LDL cholesterol (n=3665), mmol/L	3.06 (0.11)	3.02 (0.10)	3.01 (0.10)	0.48
Triglyceride (n=3661), mmol/L	1.75 (0.16)	1.72 (0.15)	1.61 (0.15)	0.007
**Taking antihypertensive medication**				
Fasting glucose (n=10 120), mmol/L	6.93 (0.15)	6.86 (0.14)	6.80 (0.14)	0.02
HbA1c (n=3262), %	6.45 (0.12)	6.28 (0.11)	6.24 (0.11)	0.007
Total cholesterol (n=9873), mmol/L	4.70 (0.10)	4.66 (0.09)	4.68 (0.09)	0.61
HDL cholesterol (n=10 154), mmol/L	1.39 (0.04)	1.40 (0.04)	1.43 (0.04)	<0.001
LDL cholesterol (n=10 188), mmol/L	2.68 (0.09)	2.69 (0.08)	2.70 (0.08)	0.39
Triglyceride (n=10 174), mmol/L	1.63 (0.11)	1.53 (0.10)	1.40 (0.10)	<0.001

Abbreviation: HbA1c, glycated Hemoglobin A1c; HDL, high-density lipoprotein; LDL: low-density lipoprotein;

*The least squares (mean ± SE) was estimated using general linear model with adjustment of age (continuous), and sex (male, or female), education attainment (less than high school, high school or equivalent, or college or above), drinking status (never drinker, former drinker, or current drinker), hypertension duration (≤5, >5 to ≤10, or >10 years), self-reported physician diagnosed CVD (yes, or no), and diabetes (yes, or no), uses of hypoglycemic (yes, or no) and lipid-lowing medication (yes, or no), systolic blood pressure (continuous), diastolic blood pressure (continuous), fasting glucose (continuous), HDL cholesterol (continuous), and eGFR (continuous).

© 2022 Lu Q et al. *JAMA Network Open.*

eTable 2. Baseline Characteristics for Participants Included and Excluded

**Characteristics**	**Included**	**Excluded**	***P***
Numbers	14 392	2916	
Age, years	65.6 (7.4)	65.9 (8.3)	0.036
Male	7115 (49.4)	1242 (43.3)	<0.001
Education attainment			
Less than high school	9497 (66.4)	1713 (60.5)	<0.001
High school or equivalent	3311 (23.2)	742 (26.2)	
College or above	1492 (10.4)	377 (13.3)	
Alcohol consumption			<0.001
Non-drinker	10 006 (69.6)	2085 (73.5)	
Current drinker	3310 (23.0)	506 (17.9)	
Former drinker	1061 (7.4)	244 (8.6)	
Duration of hypertension			<0.001
≤3 years	4646 (33.8)	773 (28.8)	
3-10 years	2771 (20.1)	585 (21.8)	
>10 years	6339 (46.1)	1329 (49.5)	
Self-reported diabetes	4026 (28.0)	769 (26.8)	0.20
Self-reported CVD	4476 (31.1)	1099 (38.3)	<0.001
Use of hypoglycemic medication	2285 (15.9)	505 (17.6)	0.022
Use of lipid-lowing medication	3600 (25.0)	755 (26.3)	0.143
Systolic blood pressure, mmHg	144 (20)	141 (20)	<0.001
Diastolic blood pressure, mmHg	83 (12)	82 (12)	0.026
Fasting glucose, mmol/L	6.3 (1.9)	6.3 (1.8)	0.56
HDL cholesterol, mmol/L	1.4 (0.4)	1.4 (0.4)	0.62
eGFR	78.8 (27.1)	78.5 (26.4)	0.58

Abbreviation: CVD, cardiovascular disease; HDL, high-density lipoprotein; eGFR, estimated glomerular filtration rate; Data are presented as mean (SD) for continuous variable or n (%) for categorical variables.

© 2022 Lu Q et al. *JAMA Network Open.*

eTable 3. HRs (95% CIs) of All-Cause and Cause-Specific Mortality According to Lifestyle Score Stratified by Antihypertensive Medication Use

	Lifestyle score				One-point increment	*P* for interaction*
	0-4 points	5-7 points	8-10 points	*P* trend		
**Not taking antihypertensive medication**						
Person-years	1606	13 764	11 437			
No. participants	236	1938	1594			
All causes mortality						0.23
No. death	63	243	138			
Model 1	1	0.49 (0.37-0.65)	0.34 (0.25-0.46)	<0.001	0.82 (0.77-0.86)	
Model 2	1	0.48 (0.36-0.63)	0.35 (0.25-0.47)	<0.001	0.83 (0.78-0.88)	
Model 3	1	0.48 (0.37-0.64)	0.35 (0.26-0.48)	<0.001	0.83 (0.78-0.88)	
CVD mortality						0.86
No. death	21	87	56			
Model 1	1	0.51 (0.32-0.82)	0.37 (0.22-0.61)	<0.001	0.83 (0.76-0.92)	
Model 2	1	0.49 (0.30-0.79)	0.38 (0.23-0.64)	0.001	0.85 (0.77-0.93)	
Model 3	1	0.53 (0.32-0.86)	0.40 (0.24-0.67)	0.002	0.85 (0.77-0.93)	
Cancer mortality						0.15
No. death	22	60	43			
Model 1	1	0.35 (0.21-0.57)	0.30 (0.18-0.52)	0.001	0.80 (0.72-0.89)	
Model 2	1	0.35 (0.21-0.57)	0.33 (0.19-0.56)	0.002	0.82 (0.73-0.91)	
Model 3	1	0.35 (0.21-0.58)	0.33 (0.19-0.57)	0.003	0.82 (0.73-0.91)	
**Taking antihypertensive medication**						
Person-years	4694	41 943	38 142			
No. participants	637	5305	4682			

© 2022 Lu Q et al. *JAMA Network Open.*

All causes mortality						-
No. death	163	855	553			
Model 1	1	0.60 (0.51-0.71)	0.43 (0.36-0.51)	<0.001	0.86 (0.83-0.88)	
Model 2	1	0.62 (0.52-0.73)	0.46 (0.38-0.55)	<0.001	0.86 (0.84-0.89)	
Model 3	1	0.62 (0.52-0.73)	0.46 (0.38-0.55)	<0.001	0.86 (0.84-0.89)	
CVD mortality						-
No. death	60	60	553			
Model 1	1	0.63 (0.48-0.83)	0.44 (0.33-0.59)	<0.001	0.85 (0.81-0.89)	
Model 2	1	0.65 (0.49-0.85)	0.46 (0.34-0.62)	<0.001	0.85 (0.81-0.90)	
Model 3	1	0.65 (0.49-0.86)	0.47 (0.35-0.63)	<0.001	0.86 (0.81-0.90)	
Cancer mortality						-
No. death	44	206	150			
Model 1	1	0.53 (0.38-0.73)	0.43 (0.30-0.60)	<0.001	0.86 (0.81-0.91)	
Model 2	1	0.54 (0.39-0.75)	0.46 (0.33-0.65)	<0.001	0.87 (0.82-0.93)	
Model 3	1	0.54 (0.39-0.75)	0.46 (0.33-0.65)	<0.001	0.87 (0.82-0.93)	

Abbreviations: CI, confidence interval; CVD, cardiovascular disease; HR, hazard ratio; **Model 1:** adjusted for age (continuous), and sex (male/ female); **Model 2:** further adjusted for education attainment (less than high school, high school or equivalent, or college or above), drinking status (never drinker, former drinker, or current drinker), hypertension duration (≤5, >5 to ≤10, or >10 years), self-reported physician-diagnosed CVD (yes/no) and diabetes (yes/no), uses of hypoglycemic (yes/no) and lipid-lowing medication (yes/no); **Model 3:** further adjusted for systolic blood pressure (continuous), diastolic blood pressure (continuous), fasting glucose (continuous), HDL cholesterol (continuous), and eGFR (continuous).

*Interaction between hypertensive medication use (yes, or no) and lifestyle (favorable, intermediate, or unfavorable), adjusted for model 3.

© 2022 Lu Q et al. *JAMA Network Open.*

eTable 4. HRs (95% CIs) for 1-Point Increase of Lifestyle Score According to Different Combination of Lifestyle Factors*

	All-cause mortality	CVD mortality	Cancer mortality
Diet, BMI, smoking status†	0.92 (0.89-0.95)	0.93 (0.88-0.99)	0.85 (0.80-0.92)
Above three plus physical activity†	0.88 (0.85-0.90)	0.89 (0.84-0.93)	0.86 (0.81-0.91)
Above four plus sleep	0.86 (0.83-0.88)	0.85 (0.82-0.89)	0.86 (0.81-0.91)

Abbreviations: BMI: body mass index; CVD, cardiovascular disease; CI, confidence interval; HR, hazard ratio;

Adjusted for age (continuous), sex (male/female), education attainment (less than high school, high school or equivalent, or college or above), drinking status (never drinker, former drinker, or current drinker), hypertension duration (≤5, >5 to ≤10, or >10 years), self-reported physician-diagnosed CVD (yes/no) and diabetes (yes/no), uses of antihypertensive (yes/no), hypoglycemic (yes/no), lipid-lowing medication (yes/no), systolic blood pressure (continuous), diastolic blood pressure (continuous), fasting glucose (continuous), HDL cholesterol (continuous), and eGFR (continuous);

†Each lifestyle factor was further adjusted when they were not included in the lifestyle score.

© 2022 Lu Q et al. *JAMA Network Open.*

eTable 5. Association of Unweighted and Weighted Lifestyle Score With the Risk of All-Cause, CVD, and Cancer Mortality

	**All-cause mortality**	**CVD mortality**	**Cancer mortality**
**Lifestyle score (unweighted)**			
0-4 (unfavorable lifestyle)	1.00	1.00	1.00
5-7 (intermediate lifestyle)	0.58 (0.50-0.67)	0.62 (0.48-0.78)	0.48 (0.37-0.63)
8-10 (favorable lifestyle)	0.43 (0.37-0.50)	0.45 (0.35-0.58)	0.42 (0.31-0.56)
**Lifestyle score (weighted)**			
First tertile (unfavorable lifestyle)	1.00	1.00	1.00
Second tertile (intermediate lifestyle)	0.64 (0.58-0.72)	0.68 (0.57-0.80)	0.63 (0.51-0.78)
Third tertile (favorable lifestyle)	0.52 (0.46-0.58)	0.49 (0.41-0.59)	0.57 (0.46-0.70)

Adjusted for age (continuous), sex (male/female), education attainment (less than high school, high school or equivalent, or college or above), drinking status (never drinker, former drinker, or current drinker), hypertension duration (≤5, >5 to ≤10, or >10 years), self-reported physician-diagnosed CVD (yes/no) and diabetes (yes/no), uses of antihypertensive (yes/no), hypoglycemic (yes/no), lipid-lowing medication (yes/no), systolic blood pressure (continuous), diastolic blood pressure (continuous), fasting glucose (continuous), HDL cholesterol (continuous), and eGFR (continuous).

© 2022 Lu Q et al. *JAMA Network Open.*

eTable 6. Associations of Different Lifestyle Score Consisting of 4 Lifestyle Factors with Risks of Mortality

Omit one lifestyle factor from the score	Lifestyle score		
	0-4	5-6	7-8
**Omit diet**			
All-cause mortality	1.00	0.65 (0.58-0.73)	0.48 (0.42-0.54)
CVD mortality	1.00	0.59 (0.49-0.71)	0.44 (0.35-0.53)
Cancer mortality	1.00	0.64 (0.51-0.81)	0.48 (0.37-0.61)
**Omit smoking status**			
All-cause mortality	1.00	0.67 (0.61-0.74)	0.66 (0.58-0.75)
CVD mortality	1.00	0.60 (0.51-0.71)	0.69 (0.56-0.84)
Cancer mortality	1.00	0.79 (0.64-0.97)	0.76 (0.59-0.98)
**Omit sleep duration**			
All-cause mortality	1.00	0.69 (0.63-0.77)	0.65 (0.57-0.74)
CVD mortality	1.00	0.70 (0.59-0.83)	0.72 (0.59-0.88)
Cancer mortality	1.00	0.67 (0.55-0.82)	0.59 (0.46-0.76)
**Omit physical activity**			
All-cause mortality	1.00	0.74 (0.67-0.82)	0.66 (0.58-0.76)
CVD mortality	1.00	0.73 (0.62-0.86)	0.73 (0.59-0.91)
Cancer mortality	1.00	0.70 (0.58-0.86)	0.56 (0.42-0.73)
**Omit BMI**			
All-cause mortality	1.00	0.65 (0.59-0.73)	0.52 (0.46-0.58)
CVD mortality	1.00	0.67 (0.56-0.80)	0.53 (0.44-0.65)
Cancer mortality	1.00	0.66 (0.53-0.81)	0.59 (0.46-0.75)

Adjusted for age (continuous), sex (male/female), education attainment (less than high school, high school or equivalent, or college or above), drinking status (never drinker, former drinker, or current drinker), hypertension duration (≤5, >5 to ≤10, or >10 years), self-reported physician-diagnosed CVD (yes/no) and diabetes (yes/no), uses of antihypertensive (yes/no), hypoglycemic (yes/no), lipid-lowing medication (yes/no), systolic blood pressure (continuous), diastolic blood pressure (continuous), fasting glucose (continuous), HDL cholesterol (continuous), and eGFR (continuous). The omitted lifestyle factor was additionally included in the model.

© 2022 Lu Q et al. *JAMA Network Open.*

eTable 7. HRs (95% CIs) for All-Cause and Cause-Specific Mortality by Antihypertensive Medication Use and Lifestyle Score After Excluding Participants With CVD at Baseline

	**Not taking antihypertensive medication**			**Taking antihypertensive medication**		
	0-4 points	5-7 points	8-10 points	0-4 points	5-7 points	8-10 points
Person-years	1241	10 755	9245	2891	27 183	25 582
No. participants	183	1506	1291	383	3421	3132
All-causes mortality						
No. death	46	154	91	75	435	300
Model 1	1	0.46 (0.33-0.63)	0.32 (0.23-0.46)	0.72 (0.50-1.04)	0.44 (0.33-0.60)	0.34 (0.25-0.46)
Model 2	1	0.46 (0.33-0.64)	0.33 (0.23-0.47)	0.68 (0.47-0.99)	0.43 (0.32-0.58)	0.33 (0.24-0.46)
Model 3	1	0.46 (0.33-0.64)	0.32 (0.23-0.46)	0.68 (0.47-0.98)	0.43 (0.31-0.58)	0.33 (0.24-0.46)
CVD mortality						
No. death	13	55	34	23	152	102
Model 1	1	0.57 (0.31-1.05)	0.42 (0.22-0.80)	0.76 (0.38-1.50)	0.52 (0.29-0.91)	0.36 (0.20-0.65)
Model 2	1	0.43 (0.22-0.81)	0.43 (0.22-0.81)	0.70 (0.35-1.38)	0.49 (0.28-0.88)	0.36 (0.20-0.65)
Model 3	1	0.57 (0.31-1.05)	0.41 (0.22-0.79)	0.70 (0.35-1.39)	0.50 (0.28-0.89)	0.37 (0.21-0.67)
Cancer mortality						
No. death	16	39	38	28	127	91
Model 1	1	0.33 (0.18-0.58)	0.38 (0.21-0.68)	0.78 (0.42-1.44)	0.37 (0.22-0.63)	0.29 (0.17-0.50)

© 2022 Lu Q et al. *JAMA Network Open.*

Model 2	1	0.33 (0.18-0.59)	0.40 (0.22-0.72)	0.79 (0.42-1.46)	0.39 (0.23-0.66)	0.32 (0.19-0.56)
Model 3	1	0.34 (0.19-0.61)	0.41 (0.22-0.73)	0.79 (0.42-1.46)	0.39 (0.23-0.66)	0.32 (0.19-0.56)

Abbreviations: CVD, cardiovascular disease; HR, hazard ratio; CI, confidence interval; **Model 1:** adjusted for age (continuous), and sex (male/ female); **Model 2:** further adjusted for education attainment (less than high school, high school or equivalent, or college or above), drinking status (never drinker, former drinker, or current drinker), hypertension duration (≤5, >5 to ≤10, or >10 years), self-reported physician-diagnosed CVD (yes/no) and diabetes (yes/no), uses of hypoglycemic (yes/no) and lipid-lowing medication (yes/no); **Model 3:** further adjusted for systolic blood pressure (continuous), diastolic blood pressure (continuous), fasting glucose (continuous), HDL cholesterol (continuous), and eGFR (continuous).

© 2022 Lu Q et al. *JAMA Network Open.*

eTable 8. HRs (95% CIs) for All-Cause and Cause-Specific Mortality by Antihypertensive Medication Use and Lifestyle Score After Excluding Participants Died Within 2 Years of Follow-up

	**Not taking antihypertensive medication**			**Taking antihypertensive medication**		
	0-4 points	5-7 points	8-10 points	0-4 points	5-7 points	8-10 points
Person-years	1592	13 707	11 412	4665	41 833	38 073
No. participants	225	1896	1572	613	5207	4627
All-causes mortality						
No. death	52	201	116	139	757	498
Model 1	1	0.49 (0.36-0.66)	0.34 (0.24-0.47)	0.81 (0.59-1.11)	0.50 (0.37-0.66)	0.36 (0.27-0.48)
Model 2	1	0.47 (0.35-0.64)	0.34 (0.24-0.47)	0.70 (0.51-0.97)	0.44 (0.33-0.59)	0.33 (0.25-0.44)
Model 3	1	0.48 (0.35-0.65)	0.34 (0.25-0.47)	0.71 (0.51-0.98)	0.45 (0.34-0.60)	0.34 (0.25-0.45)
CVD mortality						
No. death	20	68	48	52	296	193
Model 1	1	0.43 (0.26-0.70)	0.35 (0.21-0.60)	0.73 (0.44-1.23)	0.47 (0.30-0.75)	0.34 (0.21-0.53)
Model 2	1	0.34 (0.20-0.58)	0.34 (0.20-0.58)	0.58 (0.34-0.98)	0.39 (0.25-0.62)	0.29 (0.18-0.46)
Model 3	1	0.42 (0.25-0.69)	0.36 (0.21-0.61)	0.61 (0.36-1.03)	0.41 (0.26-0.65)	0.30 (0.19-0.48)
Cancer mortality						
No. death	17	56	36	39	185	131
Model 1	1	0.41 (0.24-0.71)	0.32 (0.18-0.58)	0.73 (0.42-1.30)	0.39 (0.23-0.64)	0.30 (0.18-0.50)

© 2022 Lu Q et al. *JAMA Network Open.*

Model 2	1	0.41 (0.24-0.70)	0.34 (0.19-0.61)	0.72 (0.40-1.28)	0.39 (0.24-0.65)	0.32 (0.19-0.54)
Model 3	1	0.41 (0.24-0.71)	0.34 (0.19-0.61)	0.74 (0.41-1.31)	0.40 (0.24-0.66)	0.33 (0.19-0.55)

Abbreviations: CVD, cardiovascular disease; HR, hazard ratio; CI, confidence interval; **Model 1:** adjusted for age (continuous), and sex (male/ female); **Model 2:** further adjusted for education attainment (less than high school, high school or equivalent, or college or above), drinking status (never drinker, former drinker, or current drinker), hypertension duration (≤5, >5 to ≤10, or >10 years), self-reported physician-diagnosed CVD (yes/no) and diabetes (yes/no), uses of hypoglycemic (yes/no) and lipid-lowing medication (yes/no); **Model 3:** further adjusted for systolic blood pressure (continuous), diastolic blood pressure (continuous), fasting glucose (continuous), HDL cholesterol (continuous), and eGFR (continuous).

© 2022 Lu Q et al. *JAMA Network Open.*

eTable 9. HRs (95% CIs) for All-Cause and Cause-Specific Mortality by Antihypertensive Medication Use and Lifestyle Score Among Participants With Complete Data

	**Not taking antihypertensive medication**			**Taking antihypertensive medication**		
	0-4 points	5-7 points	8-10 points	0-4 points	5-7 points	8-10 points
Person-years	1461	12 541	10 549	4126	37 122	33 936
No. participants	213	1784	1488	563	4734	4195
All-causes mortality						
No. deaths	53	229	132	142	764	489
Model 1	1	0.55 (0.41-0.74)	0.38 (0.28-0.52)	0.88 (0.64-1.20)	0.54 (0.41-0.71)	0.38 (0.28-0.50)
Model 2	1	0.54 (0.40-0.72)	0.38 (0.28-0.53)	0.75 (0.55-1.04)	0.48 (0.36-0.63)	0.35 (0.26-0.46)
Model 3	1	0.55 (0.41-0.74)	0.39 (0.28-0.54)	0.76 (0.55-1.05)	0.48 (0.36-0.64)	0.35 (0.26-0.47)
CVD mortality						
No. deaths	18	83	52	51	296	197
Model 1	1	0.59 (0.35-0.98)	0.43 (0.25-0.74)	0.87 (0.51-1.49)	0.58 (0.36-0.94)	0.35 (0.26-0.46)
Model 2	1	0.42 (0.24-0.72)	0.42 (0.24-0.72)	0.68 (0.40-1.17)	0.47 (0.29-0.77)	0.35 (0.22-0.58)
Model 3	1	0.58 (0.35-0.97)	0.44 (0.26-0.75)	0.73 (0.42-1.26)	0.50 (0.31-0.82)	0.38 (0.23-0.62)
Cancer mortality						
No. deaths	17	59	43	42	182	126
Model 1	1	0.44 (0.26-0.75)	0.39 (0.22-0.68)	0.84 (0.48-1.48)	0.41 (0.25-0.67)	0.31 (0.19-0.52)

© 2022 Lu Q et al. *JAMA Network Open.*

Model 2	1	0.45 (0.26-0.77)	0.41 (0.23-0.72)	0.84 (0.47-1.48)	0.42 (0.25-0.69)	0.33 (0.20-0.56)
Model 3	1	0.46 (0.27-0.78)	0.42 (0.24-0.74)	0.84 (0.48-1.49)	0.42 (0.25-0.70)	0.34 (0.20-0.57)

Abbreviations: CVD, cardiovascular disease; HR, hazard ratio; CI, confidence interval; **Model 1:** adjusted for age (continuous), and sex (male/ female); **Model 2:** further adjusted for education attainment (less than high school, high school or equivalent, or college or above), drinking status (never drinker, former drinker, or current drinker), hypertension duration (≤5, >5 to ≤10, or >10 years), self-reported physician-diagnosed CVD (yes/no) and diabetes (yes/no), uses of hypoglycemic (yes/no) and lipid-lowing medication (yes/no); **Model 3:** further adjusted for systolic blood pressure (continuous), diastolic blood pressure (continuous), fasting glucose (continuous), HDL cholesterol (continuous), and eGFR (continuous).

© 2022 Lu Q et al. *JAMA Network Open.*

eTable 10. HRs (95% CIs) for All-Cause and Cause-Specific Mortality by Antihypertensive Medication Use and Lifestyle Score With Further Adjustment for Mental Stress

	**Not taking antihypertensive medication**			**Taking antihypertensive medication**		
	0-4 points	5-7 points	8-10 points	0-4 points	5-7 points	8-10 points
Person-years	905	7354	6061	3209	30 756	28 531
No. participants	104	775	627	358	3276	2959
All-causes mortality						
No. death	39	143	85	110	681	465
Model 1	1	0.47 (0.33-0.67)	0.35 (0.24-0.51)	0.62 (0.43-0.90)	0.43 (0.31-0.59)	0.33 (0.24-0.46)
Model 2	1	0.47 (0.33-0.67)	0.35 (0.24-0.51)	0.62 (0.43-0.90)	0.43 (0.31-0.59)	0.33 (0.24-0.47)
CVD mortality						
No. death	11	47	33	38	258	181
Model 1	1	0.53 (0.27-1.03)	0.43 (0.22-0.86)	0.66 (0.33-1.30)	0.51 (0.28-0.94)	0.33 (0.24-0.47)
Model 2	1	0.43 (0.22-0.86)	0.43 (0.22-0.86)	0.66 (0.33-1.29)	0.51 (0.28-0.93)	0.40 (0.22-0.75)
Cancer mortality						
No. death	13	26	24	25	160	125
Model 1	1	0.27 (0.14-0.52)	0.32 (0.16-0.64)	0.49 (0.25-0.97)	0.35 (0.19-0.61)	0.31 (0.17-0.56)
Model 2	1	0.26 (0.13-0.52)	0.32 (0.16-0.64)	0.49 (0.25-0.97)	0.35 (0.20-0.62)	0.32 (0.18-0.57)

Abbreviations: CVD, cardiovascular disease; HR, hazard ratio; CI, confidence interval; **Model 1:** adjusted for age (continuous), and sex (male/ female); **Model 2:** further adjusted for education attainment (less than high school, high school or equivalent, or college or above), drinking status

© 2022 Lu Q et al. *JAMA Network Open.*

(never drinker, former drinker, or current drinker), hypertension duration (≤5, >5 to ≤10, or >10 years), self-reported physician-diagnosed CVD (yes/no) and diabetes (yes/no), uses of hypoglycemic (yes/no) and lipid-lowing medication (yes/no); **Model 3:** further adjusted for systolic blood pressure (continuous), diastolic blood pressure (continuous), fasting glucose (continuous), HDL cholesterol (continuous), and eGFR (continuous).

© 2022 Lu Q et al. *JAMA Network Open.*

eTable 11. HRs (95% CIs) for All-Cause and Cause-Specific Mortality by Antihypertensive Medication Use and Lifestyle Score When Using Asian BMI Cutoff

	**Not taking antihypertensive medication**			**Taking antihypertensive medication**		
	0-4 points	5-7 points	8-10 points	0-4 points	5-7 points	8-10 points
Person-years	2078	14 879	9850	6411	45 510	32 857
No. participants	307	2090	1371	854	5746	4,024
All-causes mortality						
No. deaths	75	256	113	197	902	472
Model 1	1	0.50 (0.39-0.65)	0.34 (0.26-0.46)	0.76 (0.58-0.99)	0.50 (0.39-0.63)	0.37 (0.29-0.47)
Model 2	1	0.50 (0.38-0.64)	0.36 (0.27-0.48)	0.67 (0.51-0.87)	0.45 (0.36-0.58)	0.35 (0.27-0.45)
Model 3	1	0.50 (0.38-0.64)	0.35 (0.26-0.48)	0.66 (0.51-0.87)	0.45 (0.35-0.57)	0.35 (0.27-0.45)
CVD mortality						
No. deaths	26	93	45	69	343	185
Model 1	1	0.52 (0.34-0.81)	0.38 (0.23-0.62)	0.72 (0.46-1.13)	0.51 (0.34-0.77)	0.35 (0.27-0.45)
Model 2	1	0.39 (0.24-0.63)	0.39 (0.24-0.63)	0.59 (0.37-0.93)	0.44 (0.29-0.66)	0.35 (0.23-0.52)
Model 3	1	0.53 (0.34-0.82)	0.40 (0.25-0.65)	0.62 (0.39-0.98)	0.46 (0.31-0.69)	0.36 (0.24-0.56)
Cancer mortality						
No. deaths	24	68	33	57	219	124
Model 1	1	0.42 (0.26-0.66)	0.31 (0.18-0.53)	0.72 (0.44-1.16)	0.39 (0.26-0.60)	0.31 (0.20-0.48)

© 2022 Lu Q et al. *JAMA Network Open.*

Model 2	1	0.43 (0.27-0.68)	0.34 (0.20-0.57)	0.71 (0.44-1.14)	0.40 (0.26-0.61)	0.34 (0.21-0.53)
Model 3	1	0.43 (0.27-0.68)	0.34 (0.20-0.57)	0.69 (0.43-1.13)	0.39 (0.26-0.60)	0.33 (0.21-0.52)

Abbreviations: CVD, cardiovascular disease; HR, hazard ratio; CI, confidence interval; **Model 1:** adjusted for age (continuous), and sex (male/ female); **Model 2:** further adjusted for education attainment (less than high school, high school or equivalent, or college or above), drinking status (never drinker, former drinker, or current drinker), hypertension duration (≤5, >5 to ≤10, or >10 years), self-reported physician-diagnosed CVD (yes/no) and diabetes (yes/no), uses of hypoglycemic (yes/no) and lipid-lowing medication (yes/no); **Model 3:** further adjusted for systolic blood pressure (continuous), diastolic blood pressure (continuous), fasting glucose (continuous), HDL cholesterol (continuous), and eGFR (continuous).

© 2022 Lu Q et al. *JAMA Network Open.*
